# Electrostatics Explains the Reverse Lewis Acidity of BH_3_ and Boron Trihalides: Infrared Intensities and a Relative Energy Gradient (REG) Analysis of IQA Energies

**DOI:** 10.1021/acs.jpca.1c05766

**Published:** 2021-09-22

**Authors:** Leonardo J. Duarte, Wagner E. Richter, Roy E. Bruns, Paul L. A. Popelier

**Affiliations:** † Chemistry Institute, University of Campinas, Campinas 13083-861, São Paulo, Brazil; ‡ Department of Chemical Engineering, Federal University of Technology—Paraná, Ponta Grossa 84017-220, Paraná, Brazil; § Manchester Institute of Biotechnology (MIB), 131 Princess Street, Manchester M1 7DN, Great Britain; ∥ Department of Chemistry, University of Manchester, Oxford Road, Manchester M13 9PL, Great Britain

## Abstract

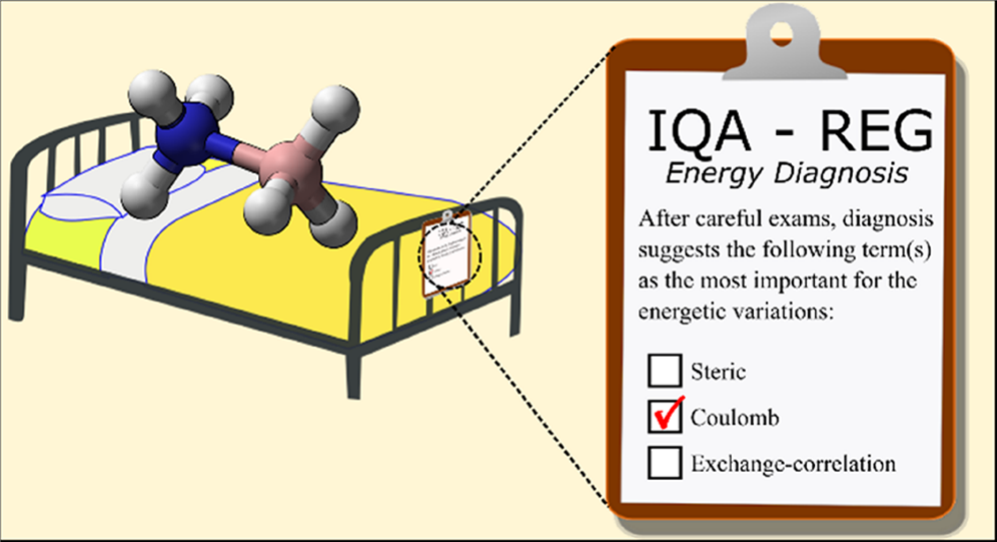

The reaction path for the formation of BX_3_–NH_3_ (X = H, F, Cl, Br) complexes was divided into two processes: (i) rehybridization of the acid while adopting a pyramidal geometry, and (ii) the complex formation from the pyramidal geometries of the acid and base. The interacting quantum atom (IQA) method was used to investigate the Lewis acidity trend of these compounds. This topological analysis suggests that the boron–halogen bond exhibits a considerable degree of ionicity. A relative energy gradient (REG) analysis on IQA energies indicates that the acid–base complex formation is highly dependent on electrostatic energy. With increasing halogen electronegativity, a higher degree of ionicity of the B–X is observed, causing an increase in the absolute value of X and B charges. This increases not only the attractive electrostatic energy between the acid and base but also enhances the repulsive energy. The latter is the main factor behind the acidity trend exhibited by trihalides. Changes in geometry are relevant only for complexes where BH_3_ acts as an acid, where lower steric hindrance facilitates the adoption of the pyramidal geometry observed in the complex. The CCTDP analysis shows that infrared intensities of BX_3_–NH_3_ are determined mostly by the atomic charges and not by the charge transfer or polarization. The opposite is observed in covalent analogues.

## 1 Introduction

An intriguing fact in inorganic chemistry is the reverse acidity of boron trihalides with respect to a strong base such as NH_3_. Naively, one would predict BF_3_ to be the strongest Lewis acid on the grounds that fluorine is the most electronegative halogen. This simple argument leads to the following wrong Lewis acidity trend: BBr_3_ < BCl_3_ < BF_3_. However, the acidity of boron trihalides unexpectedly follows the opposite trend: BF_3_ < BCl_3_ < BBr_3_. The most accepted explanation for this behavior, presented in undergraduate-level inorganic chemistry textbooks,^1,2^ invokes π-backbonding, where p occupied orbitals from the halides overlapping with the boron’s empty p orbital. This effect confers some double-bond character to the B–X bond. According to this explanation, the π-backbonding effect is more pronounced on BF_3_ because the orbital overlap is more efficient when the overlapping orbitals are similar in size and energy. During the formation of a donor–acceptor complex, such as BX_3_–NH_3_ (X = F, Cl, Br), the BX_3_ moiety will adopt a pyramidal geometry. The stronger the π-backbonding effect, the more difficult the adoption of a pyramidal geometry explaining the reverse acidity order.

In 1991, Branchadell and Oliva^3^ noted that with the increase of halogen electronegativity there is an increase in the ionic character of the B–X bond. When going from BF_3_ to BBr_3_, a reduction of σ population and a decrease of B–X polarity is observed. Also, calculations^4^ demonstrated that the p(π) overlap between B and X orbitals is larger when X = Cl compared to when X = F, which is against the π-backbonding explanation. In conclusion, B–F has a non-negligible ionic character while B–Cl and B–Br are more covalent. The ionic character of trihalides plays an important role in determining the Lewis acidity of these compounds. Subsequently, Brinck and co-workers^4^ proposed an alternative explanation, where the acidity trend was explained by the charge capacities, a measure of the ability of receiving or donating electronic charge expressed as the inverse of the difference between the ionization potential and the electron affinity, which increases in the order BF_3_ < BCl_3_ < BBr_3_. Yet, in 1999, Hirao et al.^5^ conducted a molecular orbital study where they found that the localizability of molecular orbitals on boron is important for the formation of a σ bond with a Lewis base. However, such localizability is similar for all of the trihalides, and the π-backbonding effect is not sufficient to predict the acidity trend of these compounds and the polarizability of boron must also be considered.

Gillespie^6^ also presented strong arguments in favor of the ionicity of BF_3_. Considering boron’s high charge (∼2.5 e) according to the quantum theory of atoms in molecules^7,8^ (QTAIM), a fully ionic model better explains the BF_3_ behavior and structure although this compound presents itself in a gas phase under normal conditions. The gas-phase properties of an ionic compound are explained by the Gillespie ligand close packing model.^9,10^ It shows that size limitations on boron prevent increasing its coordination number such that the formation of a solid structure is not possible.^11^

Rejecting Gillespie’s interpretation, Haaland et al.^12^ claimed that the QTAIM charges are not reliable because the atomic polar tensor (APT) approach assigns boron an atomic charge of almost one unit lower than Gillespie’s value. However, Haaland’s observation is not precise because an APT “charge” contains atomic polarizations,^13^ and QTAIM polarizations should be included to obtain a valid comparison.

In this work, we present a novel approach to understanding the unexpected Lewis acidity scale of boron trihalides using the recently proposed relative energy gradient (REG)^14^ method combined with a topological energy partition scheme called the interacting quantum atoms (IQA).^15^ We also performed an infrared intensity analysis on the acid–base complexes by means of the charge–charge transfer-dipolar polarization (CCTDP)^16^ model. This model was applied to the out-of-plane bending modes given that rehybridization (from sp^2^ to sp^3^) with accompanying pyramidalization is expected to affect acidity behavior. Considering the topological nature of QTAIM, on which our methods are based, we will present and develop a new hypothesis based only on the electronic density properties, which is independent of the orbital concept and, therefore, independent of the mainstream π-backbonding effect.

## 2 Background and Methodology

### 2.1 IQA Energy Partitioning Scheme

The IQA partitioning scheme utilizes the QTAIM definition of atomic basins to calculate intra- and interatomic energy terms that sum to the total energy of the system. The intra-atomic terms consist of the sum of the electronic kinetic energy, the electron–electron repulsion, and electron–nucleus attractive potential inside a single atomic basin. It has been demonstrated that compression of atomic volume causes an exponential increase in the intra-atomic energy, thereby showing that intra-atomic terms act as a measure of steric effects.^17^

Interatomic terms contain the (classical) electrostatic, the exchange, and the correlation energy between two distinct atoms. The electrostatic interaction can be associated with charge transfer, polarity, and ionicity. The exchange term relates the purely quantum mechanical effect of electron delocalization to covalency, bond order, and (hyper)conjugation. Finally, the correlation term connects with London dispersion, which is a well-studied type of van der Waals interaction. Note that exchange and correlation terms are often added together, as a consequence of the *ansatz* of density functional theory, which is adopted in this article. We also note that the above interpretation of IQA terms results in ionicity and covalency not being opposite of each other.^18^

The total system energy is given by the sum of energies of individual atoms according to eq 1
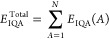
1where *N* is the total number of atoms in the system and *A* denotes an atom. Each atomic energy can be further expanded as a sum of intra- and interatomic contributions
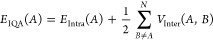
2with *V*_Inter_ (*A,B*) being the potential energy between atoms *A* and *B*.

The intra-atomic term, *E*_Intra_ (*A*), is equal to the sum of the (intra-atomic) kinetic energy, *T*(*A*), and electron–electron, *V*_ee_ (*A*), and electron–nucleus *V*_en_ (*A*) potential energies. The interatomic contributions are given by
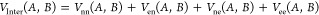
3where subscripts e and n, respectively, denote electrons and nuclei. The electron–electron potential energy encompasses two contributions: the classical Coulombic energy and the exchange-correlation components.
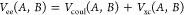
4All Coulombic terms can be grouped together into the classical potential term *V*_cl_ (*A,B*). The interatomic terms can then be written as
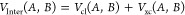
5A full description of the IQA partitioning scheme can be found elsewhere.^19,20^

### 2.2 Relative Energy Gradient (REG) Analysis^14^

The number of IQA terms quadratically increases with the number of atoms in a system. Using a resolution of three terms (i.e., intra + electrostatic + exchange-correlation), *N*^2^ terms are necessary to describe the total energy of an *N*-atom molecule. When faced with the system changing along a potential energy surface (PES), the resulting large number of IQA terms makes a manual energy analysis impractical. To help with this task, the REG method was proposed. In addition, REG is a minimal and thus unbiased method that ranks IQA terms of a PES according to the degree by which they act like the total energy of a system. To create this action, one needs to impose a dynamical change to the system, for example, a rotation around a relevant bond or the compression of a hydrogen bond. Such a change is governed by a so-called control coordinate. REG thus answers one of the basic questions of any chemical phenomenon: which atoms cause it and why? Second, REG enables the construction of a small set of energies that describe the energetic profile of the system using a control coordinate. The latter can be a dihedral angle or a hydrogen bond length. Finally, the PES is divided into energy segments that, ideally, begin and end at a stationary point in the PES.

For each (energy) segment, a least-squares regression is performed involving each IQA term and the total energy by
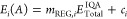
6where *E*_*i*_ (*A*) is one of the many individual intra- or interatomic energy terms, *m*_REG,*i*_ is the REG coefficient, with *i* being the index denoting the type of energy and the atom involved, and *c*_*i*_ is the intercept for the IQA contribution *i*. Note that the sum of all *c_i_* values is zero and so far no physical meaning has been attributed to *c_i_*. From eq 12 in the original REG publication,^14^ it follows that the higher the absolute value of *m*_REG*,i*_ (i.e., “REG value”) the more the dynamic behavior of the energy contribution *E*_*i*_ that contributes to that of the total system. As such, ranked REG values are a list of energy terms of decreasing importance in explaining the total system, starting with the most important one.

It can be shown^14^ that the positive REG values are IQA terms that have the same sign in energy gradient as the total system for a given segment. Put differently, the IQA terms act in the same way as the total energy does, i.e., these terms work in favor of the segment. Similarly, IQA terms with negative REG coefficients “work against” the segment. Another important quantity in a REG analysis is the Pearson coefficient, *R*, which ranges from −1 to 1. If |*R*| is very different from unity, then eq 6 becomes invalid and *m*_REG,*i*_ loses its physical meaning.

### 2.3 CCTDP Model^16^

Infrared intensities emerge from changes in the electron density that occur when atoms start moving following a vibrational normal coordinate. The infrared intensity, *A* [km mol^–1^], is proportional to the square of the molecular dipole moment, *p⃗*, differentiated with respect to the normal coordinate, *Q*
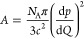
7where *c* is the speed of light and *N*_A_ is Avogadro’s constant. The dipole moment of a molecule, *p⃗*, is defined as the sum, over all atoms, of each atomic charge multiplied by its equilibrium coordinate as well as a sum over all of e atomic dipole moments. This statement is formally expressed in eq 8, where σ = *x*, *y*, or *z* denotes each component of the molecular dipole moment
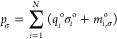
8where *q*_*i*_^o^ is the equilibrium charge of atom *i*, σ_*i*_^o^ is the equilibrium Cartesian coordinate of atom *i*, and *m*_*i*,σ_^o^ is the σth component of the *i*th equilibrium atomic dipole moment. Taking the derivative of *p*_σ_ with respect to the *j*th atomic Cartesian coordinate (*i* = 1, ..., 3*N*) results in
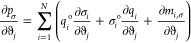
9

The terms inside the parentheses are, respectively, the charge (*C*), charge transfer (CT), and dipolar polarization (DP) contributions, which make up the parts of the acronym of the CCTDP model for dipole moment derivatives. Note that the *C* term is zero for all atoms except when σ = ϑ and *j* = *i*. The  derivative corresponds to the *p*_σϑ_ element of the atomic polar tensor (APT) of atom *i*.

Elements from the APT can be converted into terms of normal coordinates by multiplying with an appropriate L-matrix element,, as follows
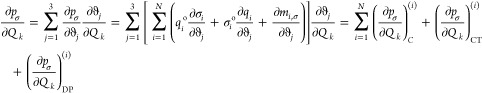
10The terms in brackets on the right-hand side of eq 10, respectively, contain the charge, charge transfer, and dipolar polarization contributions to the dipole moment derivative owing to a displacement of the *i*th atom with a magnitude specified by the normal coordinate *Q*_*k*_. Note that in eq 10 we have swapped indices *i* and *j* to obtain the final (bottom) expression, which can be further written as in eq 11, where a sum over all of the atomic displacements, *j* = 1,2,3 (*x,y,z*), has taken place
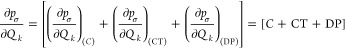
11resulting in the charge (*C*), charge transfer (CT), and dipolar polarization (DP) terms of our model.

## 3 Methodology

**Figure 1:**
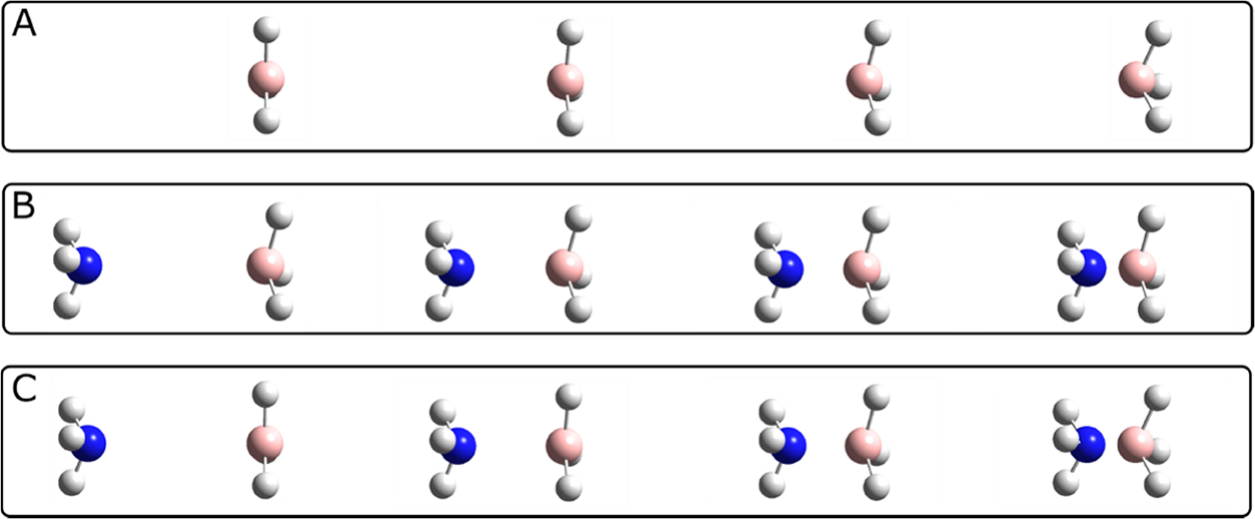
(A) Lewis acid rehybridization process, (B) geometries along the direct (constrained) complexation path, and (C) geometries along the complexation reaction path. Note that in (C), all atoms are able to move, while in (B) the monomeric geometry is maintained to that of the monomer inside the complex.

The complexation reaction can be divided into two main processes: (i) rehybridization of the Lewis acid (Figure 1A), and (ii) direct complexation (Figure 1B), i.e., the approach between the Lewis base and the distorted (i.e., pyramidal and of fixed geometry) form of the acid. Figure 1A exemplifies the rehybridization process of BX_3_ (X = H, F, Cl, or Br) where the X atoms (now also including H) are displaced out of the (original) molecular plane (left). When performing the REG analysis, for each geometry both the out-of-plane angle and the B–X distances are fixed. Figure 1B shows a series of geometries along the direct complexation path, where both moieties of the complex are brought together by decreasing the B–N distance but conserving both monomers’ geometries as they are in the equilibrium state of the complex. Figure 1C results from the two previous processes and presents a series of geometries along the complexation reaction path. The REG analysis was carried out using an in-house Python program called *REG.py.*(21)

The structures of the complexes were optimized at the MP2/aug-cc-pVTZ level of theory using GAUSSIAN09 revision D1.^22^ The counterpoise^23^ method was applied to account for the basis set superposition error (BSSE). The same level of theory was applied to the monomers for their equilibrium and out-of-equilibrium geometries and to calculate the infrared intensities following the CCTDP model using the program PLACZEK.^24^

To perform the REG analysis, a different level of theory was applied: B3LYP/6-311+G(d,p). The reason is that MP2-IQA is computationally very expensive, while B3LYP was made compatible^25^ with IQA a few years ago. Since its version 14.04.17, AIMAll introduced an important modification in the IQA formalism, which allows the recovery of *V*_xc_ (B,X) from DFT. The modification consists of calculating the interatomic exchange-correlation term using the pure Hartree–Fock exchange equation but by replacing the HF orbitals with Kohn–Sham orbitals (see eq 14 in ref (25)).

The wavefunctions for 40 points over the whole reaction path, from 1.0 to 4.9 Å, were calculated for each acid, that is, BH_3_, BF_3_, BCl_3_, and BBr_3_. IQA terms were calculated by integration over atomic basins using the AIMAll program.^26^ The same level of theory was also applied to investigate the rehybridization process.

**Figure 2:**
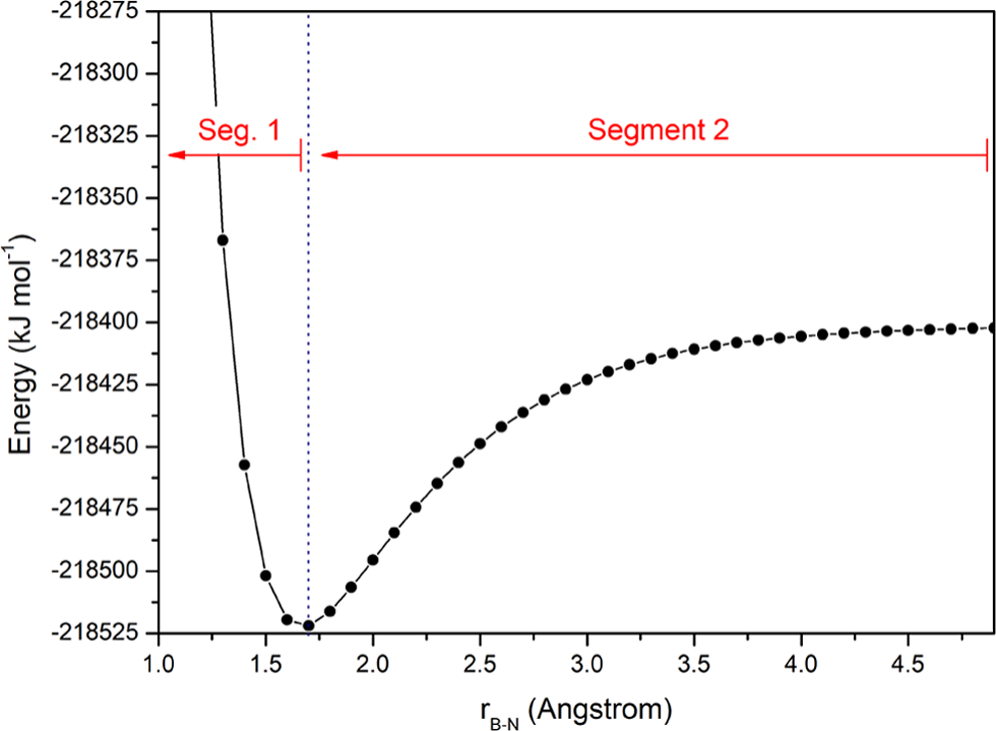
Two segments (separated by the vertical line) appearing in the PES governing the formation of the BH_3_–NH_3_ complex. Each segment is subjected to its own REG analysis. The direction of analysis is always from right to left. Note that some energies at very short range are not shown to keep the full profile in proportion.

Figure 2 shows the PES of the formation of the BH_3_–NH_3_ complex as an example. In this work, we analyze the process from right to left, i.e., in the direction of formation instead of the direction of dissociation. This choice is more intuitive to explain the REG analysis. The same direction of analysis is valid for the trihalides complexes.

## 4 Results and Discussion

### 4.1 Thermodynamics of the Complex Formation

**Figure 3:**
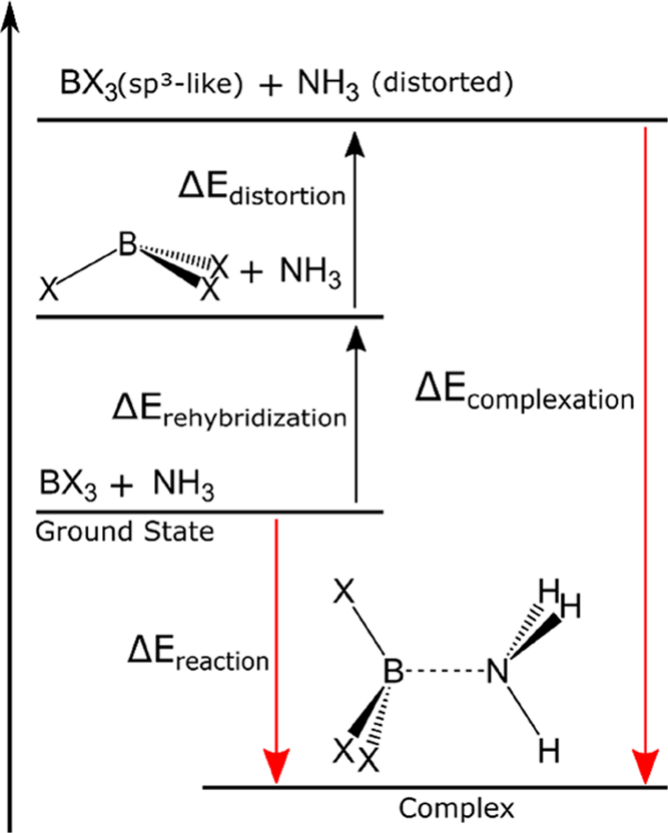
Thermodynamic cycle (not to scale) for the Lewis acid–base reaction between BX_3_ and NH_3_, where X = H, F, Cl, or Br. The red arrow on the right corresponds to the complexation path (Figure 1B), while the one on the left corresponds to the reaction path (Figure 1C). The geometries of the monomers at the top are the same as those of the monomers inside the complex.

Figure 3 presents a thermodynamic cycle for the complexation process. The quantity Δ*E*_reaction_ corresponds to the difference in energy of the complex and that of the separated monomers plus the BSSE correction, for the reaction path. This energy difference is seen as a measure of the Lewis acidity in the gas phase (Figure 1C). On the other hand, the quantity Δ*E*_complexation_ corresponds to the difference in energy of the complex and that of the distorted geometries of both the Lewis acid and base, also including the BSSE correction (Figure 1B). Next, Δ*E*_rehybridization_ is equal to the energy necessary to force the planar sp^2^ boron atom to adopt a pyramidal sp^3^-like structure (Figure 1A) and is given by the difference between the BX_3_ energy when adopting its geometry as it is in the equilibrium state of the complex and the equilibrium geometry of the isolated BX_3_ molecule. Finally, Δ*E*_distortion_ corresponds to the energy necessary for the small distortion of the Lewis basis, NH_3_, and geometry. The reaction energy can be written as
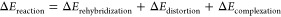
12

Table 1 contains the calculated values for each term of eq 12. Note that the absolute values of Δ*E*_reaction_ follow the experimental Lewis acidity trend, i.e., BH_3_ > BBr_3_ ≥ BCl_3_ > BF_3._ When the absolute values of the complexation energy, Δ*E*_complexation_, are considered, then the following order is found: BH_3_ ≈ BF_3_ < BCl_3_ ≈ BBr_3_.

**Table 1:** Calculated Values for Each Energy Term (kJ mol^–1^) Appearing in eq 12 at the MP2/aug-cc-pVTZ Level of Theory

molecule	Δ*E*_rehybridization_	Δ*E*_distortion_	Δ*E*_complexation_	Δ*E*_reaction_
BH_3_	54.6	0.2	–183.6	–128.8
BF_3_	97.0	0.6	–185.9	–88.3
BCl_3_	97.4	1.0	–210.5	–111.9
BBr_3_	90.4	1.1	–210.8	–119.2

The quantity Δ*E*_distortion_ corresponds to the energy changes of the Lewis base structure, which are caused by the approach of the Lewis acid. For all of the systems, Δ*E*_distortion_ is lower than 1.1 kJ mol^–1^ and can thus be neglected. In contrast, the rehybridization of the boron trihalides requires a large amount of energy. During the rehybridization process, the atoms bonded to boron are displaced out of the molecular plane causing boron to adopt an unfavorable sp^3^ geometry. The rehybridization energy required to deform BH_3_ is nearly half of the energy necessary to rehybridize the boron trihalides, that is, ∼55 instead of 90–100 kJ mol^–1^ for the halides. This fact partially explains why BH_3_ is the strongest Lewis acid presented here, which is decided by the values of Δ*E*_reaction_. Indeed, the addition of BH_3_’s much smaller positive rehybridization energy (∼55 kJ mol^–1^) to the complexation energy (∼−200 kJ mol^–1^) that is roughly similar for all compounds, results in BH_3_ having the most negative reaction energy. Although BH_3_ leads to the least stable complexation energy, from the ease of rehybridizing follows a more stable value of Δ*E*_reaction_. However, rehybridization energy alone is not enough to explain the boron trihalides’ reverse acidity because its values are very similar to the boron trihalides.

### 4.2 Rehybridization of the Lewis Acid

**Figure 4:**
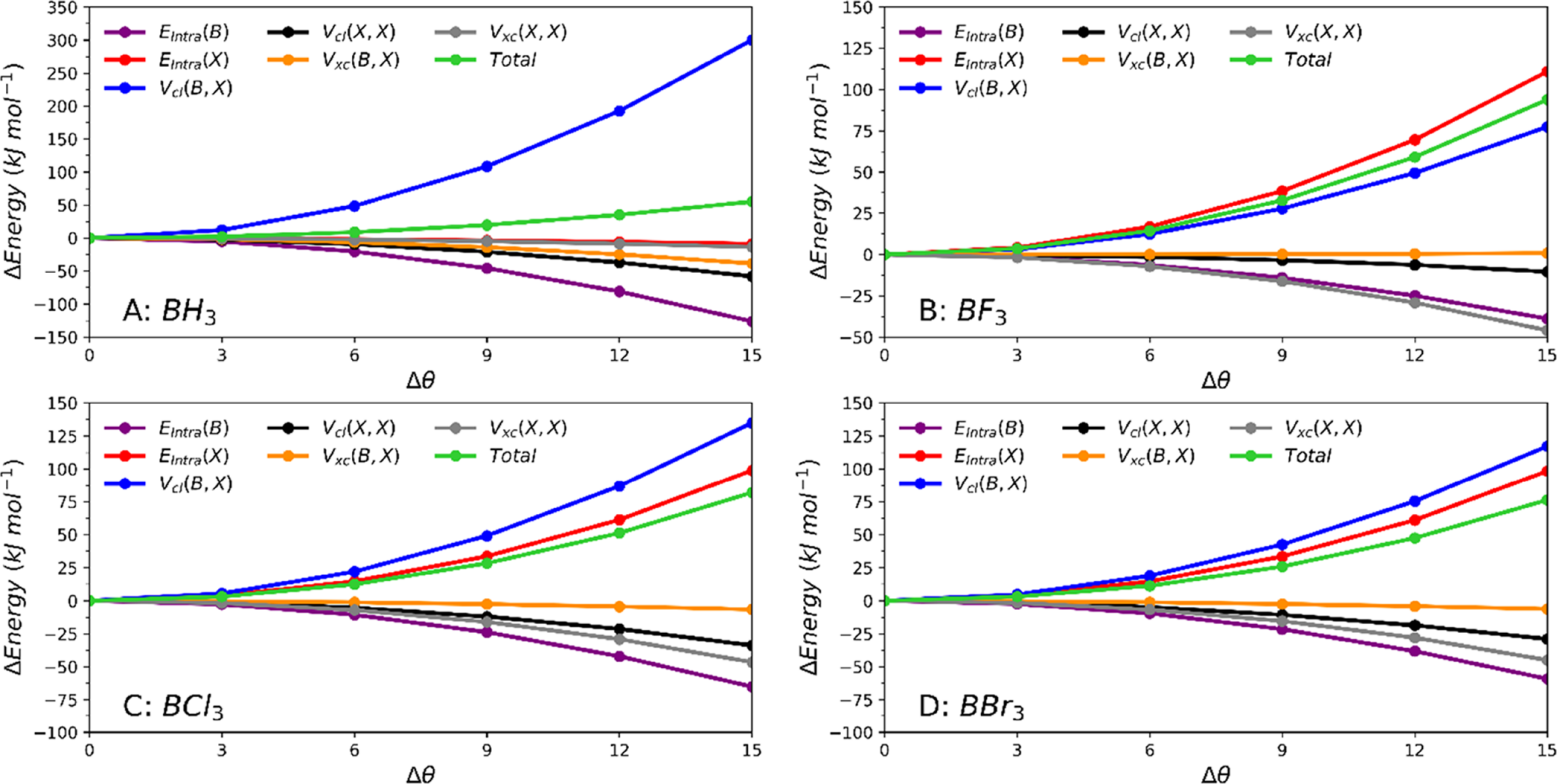
Six IQA energy contributions (and their sum) involved in the rehybridization process for BH_3_, BF_3_, BCl_3_, and BBr_3_ as a function of Δθ = θ – 90°.

To further investigate the rehybridization process, a series of IQA calculations were performed on the Lewis acids by varying the θ angle (between the BX bonds and the molecular C_3_ rotation axis) between 90 and 105°. The value of 90° corresponds to a planar BX_3_ geometry and the larger the value beyond 90°, the more pyramidal the molecule. Applying the IQA energy decomposition scheme, and grouping (i.e., adding, so the contribution of X counts as 3 times X) equivalent atoms, six terms were obtained: *E*_Intra_ (B), *E*_Intra_ (X), *V*_cl_ (B,X), *V*_cl_ (X,X), *V*_xc_ (B,X), and *V*_xc_ (X,X). The symbol X represents the sum of the contributions of all X atoms, including H, that are bonded to boron. Figure 4 shows the energy profile of these six energy terms as well as their sum (“Total”) as a function of Δθ = θ – 90°. The plots show that *V*_cl_ (B,X) is destabilizing for all systems, i.e., the energy contribution becomes less negative with increasing Δθ, which corresponds to increasing pyramidalization. In other words, the classical electrostatic interaction between B and X counters the rehybrization process (from sp^2^ to sp^3^).

Considering the constraint that the molecular charge remains constant during the rehybridization process, any change in the boron atomic charge will cause the same change, but of the opposite sign, in the charge of atom X. In that way, the (classical) electrostatic energy, *V*_cl_ (B,X), becomes a measure of the electrons’ transference between bonded atoms. The more the charge transferred, the greater the magnitude of the electrostatic energy between B and X. During the rehybridization process, the classical potential between boron and the terminal X atoms becomes more positive, i.e., it destabilizes the system, suggesting that interatomic electron transfer is not favored in the distorted energy. The total variation (going from the monomeric geometry to the rehybridized geometry of the complex) in boron’s atomic charge for BH_3_, BF_3_, BCl_3_, and BBr_3_ are, respectively, −0.05, −0.02, <−0.01, and −0.04 e (see Table S1).

As stated before, the rehybridization energy of BH_3_ is about half of the rehybridization energy on the trihalides. This can be explained by steric effects. From all of the plots in Figure 4, BH_3_ is the only molecule for which *E*_Intra_ (X) is not destabilizing. For all the others, *E*_Intra_ (X) is about as destabilizing as *V*_cl_ (B,X). The energy term *E*_Intra_ (X) is a measure of the steric hindrance that the X atoms experience. When boron trihalide is forced to adopt the pyramidal shape, the X atoms become closer to each other. This change is more pronounced in BF_3_ because the B–F bond is shorter (than other B–X bonds), thus bringing the F atoms closer for the same Δθ value.

Table 1 shows that BBr_3_ has the lowest rehybridization energy among the trihalides. While the rehybridization energy of BF_3_ is mostly determined by the increase in boron’s intra-atomic energy, the rehybridization energies of BCl_3_ and BBr_3_ are mostly determined by the repulsive electrostatic energy between B and X. The slightly higher value *V*_cl_ (B,X) in BCl_3_ increases its rehybridization energy.

### 4.3 Direct Complexation Path

The direct complexation path corresponds to the formation of the complex after the rehybridization of the Lewis acid. In fact, both the direct complexation path and the rehybridization occur simultaneously during the reaction but to understand the physical processes that drive this chemical reaction, it is convenient to break the overall reaction into smaller steps.

Because the introduction of the Lewis base NH_3_ increases the number of IQA terms, we use the REG analysis to rank the most important IQA energy contributions. The results of the REG analysis are displayed in Table 2.

**Table 2:** REG Analysis Results for the Direct Complexation Process^a^

	segment 1			segment 2		
acid	IQA term	REG	*R*^2^	IQA term	REG	*R*^2^
BH_3_	*E*_intra_ (N)	1.48	0.92	*V*_cl_ (B,N)	6.66	0.96
	*V*_cl_ (B,X)	0.70	0.99	*V*_cl_ (X,H)	4.93	0.96
	*V*_cl_ (B,H)	0.52	0.77	*V*_cl_ (B,X)	2.11	0.92
	*E*_intra_ (B)	0.37	0.89	*V*_cl_ (N,H)	2.09	0.99
	*V*_cl_ (X,N)	0.25	0.56	*V*_xc_ (B,N)	1.18	0.99
	*V*_cl_ (X,X)	–0.24	0.95	*V*_cl_ (X,X)	–0.93	0.96
	*V*_xc_ (X,N)	–0.26	0.87	*V*_xc_ (B,X)	–0.95	0.99
	*V*_xc_ (B,N)	–0.26	0.92	*E*_intra_ (N)	–1.73	0.88
	*V*_cl_ (N,H)	–0.27	0.84	*V*_cl_ (X,N)	–5.02	0.94
	*V*_cl_ (B,N)	–1.29	0.75	*V*_cl_ (B,H)	–5.57	0.97
BF_3_	*E*_intra_ (N)	1.52	0.91	*V*_cl_ (B,N)	7.82	0.96
	*V*_cl_ (B,H)	0.82	0.84	*V*_cl_ (X,H)	6.00	0.98
	*V*_cl_ (B,X)	0.75	0.96	*V*_cl_ (N,H)	2.53	0.98
	*V*_cl_ (X,N)	0.43	0.72	*V*_xc_ (X,N)	1.19	0.97
	*E*_intra_ (B)	0.17	0.82	*V*_xc_ (B,N)	0.85	0.97
	*V*_xc_ (B,N)	–0.22	0.94	*V*_xc_ (N,H)	–0.81	1.00
	*V*_cl_ (X,H)	–0.24	0.71	*E*_intra_ (H)	–0.88	1.00
	*V*_xc_ (X,N)	–0.30	0.88	*E*_intra_ (N)	–1.68	0.81
	*V*_cl_ (N,H)	–0.67	0.90	*V*_cl_ (X,N)	–5.83	0.96
	*V*_cl_ (B,N)	–1.32	0.72	*V*_cl_ (B,H)	–6.84	0.98
BCl_3_	*E*_intra_ (N)	1.70	0.94	*V*_cl_ (B,N)	7.99	0.93
	*V*_cl_ (B,H)	0.83	0.87	*V*_cl_ (X,H)	4.96	0.98
	*V*_cl_ (B,X)	0.52	1.00	*V*_cl_ (N,H)	3.45	0.97
	*E*_intra_ (B)	0.45	0.86	*V*_cl_ (B,X)	3.00	1.00
	*V*_cl_ (X,N)	0.30	0.71	*V*_xc_ (B,N)	1.36	1.00
	*V*_cl_ (X,X)	–0.16	0.96	*V*_xc_ (B,X)	–1.39	0.99
	*V*_xc_ (B,N)	–0.22	0.95	*E*_intra_ (B)	–1.61	0.82
	*V*_xc_ (X,N)	–0.26	0.89	*E*_intra_ (N)	–2.61	0.84
	*V*_cl_ (N,H)	–0.84	0.93	*V*_cl_ (X,N)	–4.86	0.97
	*V*_cl_ (B,N)	–1.59	0.81	*V*_cl_ (B,H)	–6.37	0.98
BBr_3_	*E*_intra_ (N)	1.77	0.93	*V*_cl_ (B,N)	7.35	0.92
	*V*_cl_ (B,H)	0.89	0.87	*V*_cl_ (B,X)	4.74	0.98
	*E*_intra_ (B)	0.65	0.85	*V*_cl_ (X,H)	4.22	0.99
	*V*_cl_ (X,N)	0.28	0.66	*V*_cl_ (N,H)	3.62	0.97
	*V*_xc_ (B,X)	0.22	0.83	*V*_xc_ (B,N)	1.57	1.00
	*V*_cl_ (X,X)	–0.08	0.99	*V*_xc_ (B,X)	–1.82	0.99
	*V*_xc_ (B,N)	–0.23	0.95	*E*_intra_ (N)	–2.77	0.86
	*V*_xc_ (X,N)	–0.25	0.88	*E*_intra_ (B)	–2.95	0.90
	*V*_cl_ (N,H)	–0.90	0.93	*V*_cl_ (X,N)	–4.06	0.97
	*V*_cl_ (B,N)	–1.71	0.81	*V*_cl_ (B,H)	–5.71	0.98

a The control coordinate is the B–N distance. Segment 1 corresponds to the shortening of the B–N bond beyond the equilibrium point, while Segment 2 corresponds to the formation of B–N bond. The atom X corresponds to H, F, Cl, and Br.

Note that the terms in Table 2 that involve hydrogen account for the sum of all three hydrogen atoms bonded to nitrogen. Note also that the BX_3_–NH_3_ system has 8 atoms and so there are 8^2^ = 64 IQA energy terms in total but only those with the highest absolute values for the REG coefficient are shown. We first discuss Segment 1 for which the B–N distance is always shorter than that at the equilibrium geometry. For all complexes, the most positive REG value is found for *E*_intra_ (*N*), which means that the intra-atomic energy of nitrogen contributes most to the shape of the total energy profile in Segment 1. Thus, upon compression of the complex beyond equilibrium, nitrogen’s internal energy explains best the destabilization that is experienced by the whole complex. In contrast, *V*_cl_ (B,N) has the most negative REG coefficient again for all complexes. This electrostatic energy term thus works most against the energy barrier, which is Segment 1 (when interpreted in the direction of compression). This fact makes sense if the electrostatic interaction can be truncated at the level of monopole moments, for the sake of interpretation. Indeed, because B and N are oppositely charged, the electrostatic energy decreases (i.e., becomes more negative) as the two nuclei approach each other. This stabilization substantially counters the energy profile of the whole complex.

**Figure 5:**
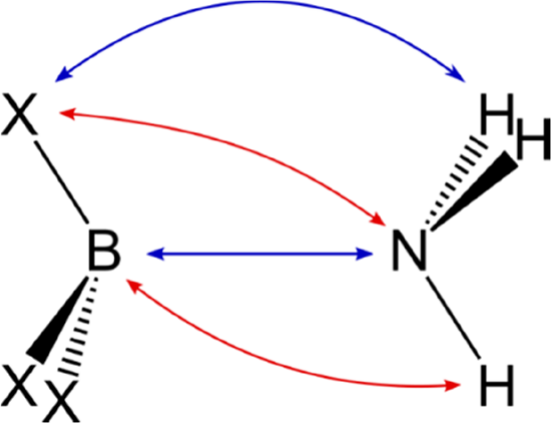
Scheme showing the attractive electrostatic interaction (blue) and the repulsive electrostatic interaction (red). Multiple instances of the same interactions are omitted to simplify the figure.

Segment 2 runs from infinity to the equilibrium distance (see Figure 2). For all four systems, *V*_cl_ (B,N) comes up as the energy term with the most positive REG value. This observation proves that the electrostatic attraction between B and N dominates and indeed “steers” the complex formation. The second most positive REG coefficient is that of *V*_cl_ (X,H), which is not surprising, except perhaps when X = H. However, the QTAIM charge of H in BH_3_ is sizeably negative (∼−0.7 e), indicative of the hydridic character of this hydrogen. Finally, *V*_cl_ (B,H) (where H belongs to NH_3_) is dominant in working against the complex formation by displaying the largest negative REG value, closely followed by *V*_cl_ (X,N). These dominant intermolecular repulsive interactions are again easy to understand as major disruptors of complex formation. Figure 5 shows these repulsive and attractive interactions, thereby summarizing the electrostatic nature of complex formation.

We now explain why an increase in charge (in absolute values) results in a more repulsive electrostatic energy between the acid and base. Based on only the electronegativity scale, one expects that the resultant attractive potential energy will follow the order BBr_3_ < BCl_3_ < BF_3_. This is because the more electronegative X, the more positive boron’s charge will be, thereby increasing the attractive potential energy between the acid and the base, i.e., *V*_cl_ (B,N). However, a chemical bond (e.g., BX) relies on one atom sharing its electrons with another. This means that a larger difference in electronegativity will not only increase the positive charge on boron but will also increase the negative charges on X atoms. This, in turn, implies that an increase in the electronegativity difference will also increase the repulsive potential between X and N and between B and H. Table 1 identifies two groups according to the complexation energy: BH_3_ and BF_3_ at around 185 kJ mol^–1^, and BCl_3_ and BBr_3_ both around 210 kJ mol^–1^. BCl_3_ and BBr_3_ have almost the same complexation energy, which is explained by similarities in charge and bond distances. Among the trihalides, BBr_3_–NH_3_ and BCl_3_–NH_3_ show the lowest absolute values of charge for the B and halide X atoms. On the other hand, they exhibit the greatest absolute charge on nitrogen. As the B–N distance is almost constant for all complexes, the lower charge on B is compensated by the greater charge on N. These compounds also present longer bond distances between B and X, which implies greater distances between the B···H and N···X repulsive pairs. Similar behavior is expressed by BH_3_NH_3_ and BF_3_NH_3_. Although similar values of Δ*E*_complexation_ are found for BH_3_–NH_3_ and BF_3_–NH_3_, they emerge for different reasons. The BF_3_–NH_3_ complexation energy is attenuated by the proximity of the repulsive pairs, while the complexation energy in BH_3_–NH_3_ is enhanced by the lower charge of the repulsive pairs. Geometrical parameters and atomic charge values are presented in Table S1.

The magnitudes of the REG values for exchange-correlation terms are always less than one-third of the REG value of the electrostatic terms. Because the REG value measures the degree to which an individual energy contribution explains the energy change in the total system, the low-ranked exchange-correlation energies perform secondary roles in describing the overall reaction process.

### 4.4 Reaction Path

The overall reaction path is the resultant of the two previous processes. The REG analysis over the reaction path shows again that the intermolecular electrostatic contributions feature among the highest-ranked IQA terms in Segment 2, while Segment 1 continues to be dominated by the steric effect of the nitrogen atom, *E*_intra_(N). Table S2 presents the results for the REG analysis over the reaction path. Once again, the balance between the attractive and repulsive electrostatic potential is the key to understanding the Lewis acidity order of boron trihalides.

**Figure 6:**
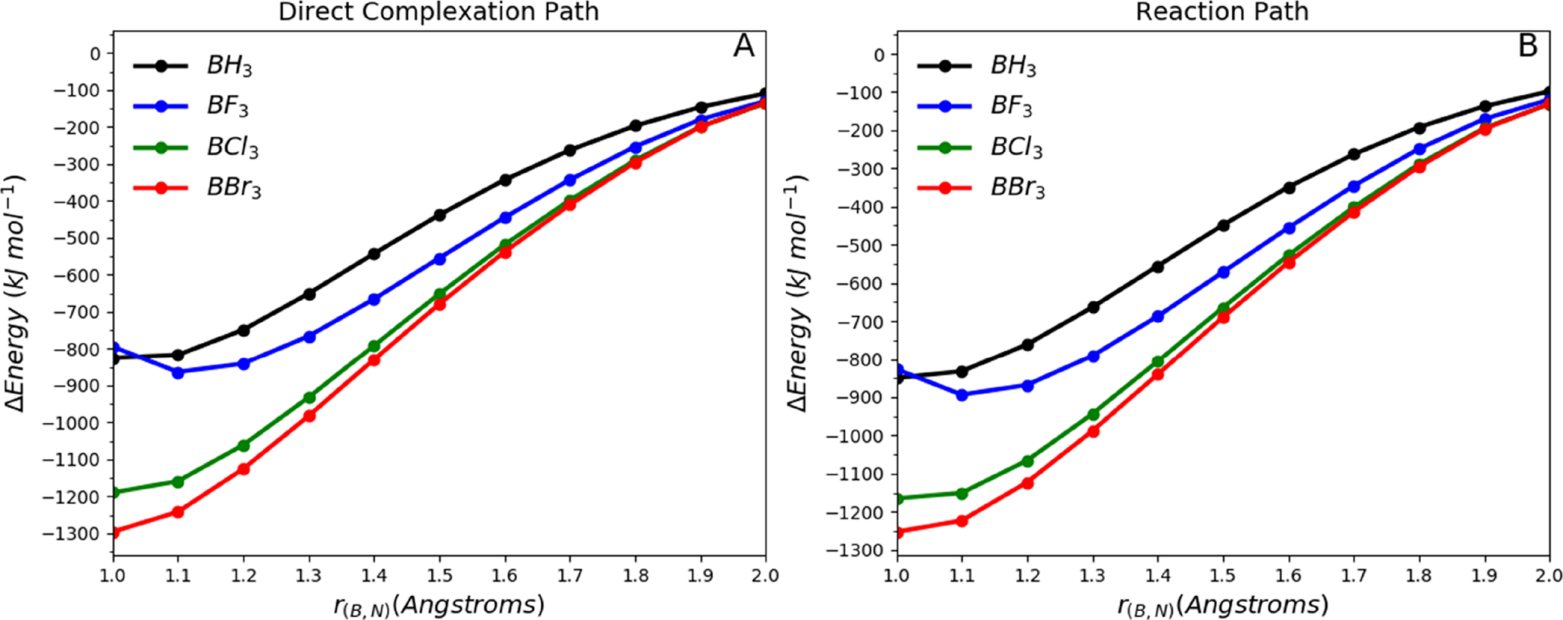
Total intermolecular electrostatic potential energies given by *V*_cl_ (B,N) + *V*_cl_ (X,H) + *V*_cl_ (X,N) + *V*_cl_ (B,H) as a function of the B–N distance. IQA contributions for (A) the direct complexation path and (B) the reaction path.

Figure 6 displays two different plots. Figure 6A shows the sum of energy terms *V*_cl_ (B,N), *V*_cl_ (X,H), *V*_cl_ (X,N), and *V*_cl_ (B,H) against the B–N distance in the direct complexation path (see Figure 1C), while Figure 6B shows the sum of those terms against the distance between B and N within the reaction coordinate. The main difference between both panels of Figure 6 is that, in the left panel, the BX_3_ and NH_3_ moieties are forced to maintain the complex’s (monomeric) geometry over the whole path while, in the right panel, all atoms can move.

For both paths, the sum of the intermolecular classical potential energies is always stabilizing (lower than zero) and follows the stabilization trend: BBr_3_ > BCl_3_ > BF_3_ > BH_3_. In fact, it is difficult to spot any difference between the two plots. This suggests that the rehybridization process, although important for the acidity order among BH_3_ and the trihalides, does not cause any significant change in the charge disposition, leaving the electrostatic potential energy largely unchanged.

The high importance of the electrostatic terms is surprising because it indicates that the B–N bonds have a high degree of ionicity. From the IQA point of view, the degree of covalency/ionicity of a molecule is not a one-dimensional measure, where a highly covalent bonded molecule has low ionicity or vice versa, but a two-dimensional^18^ scale, where a molecule can be highly covalent and show high ionicity as well. Within this approach, the B–N bond in all complexes is strongly ionic, expressed by the high magnitude of *V*_cl_ (B,N). At the same time, the exchange-correlation terms for these bonds are small, confirming the results of the REG analysis, where the classical terms are more highly ranked compared to the exchange-correlation terms.

**Figure 7:**
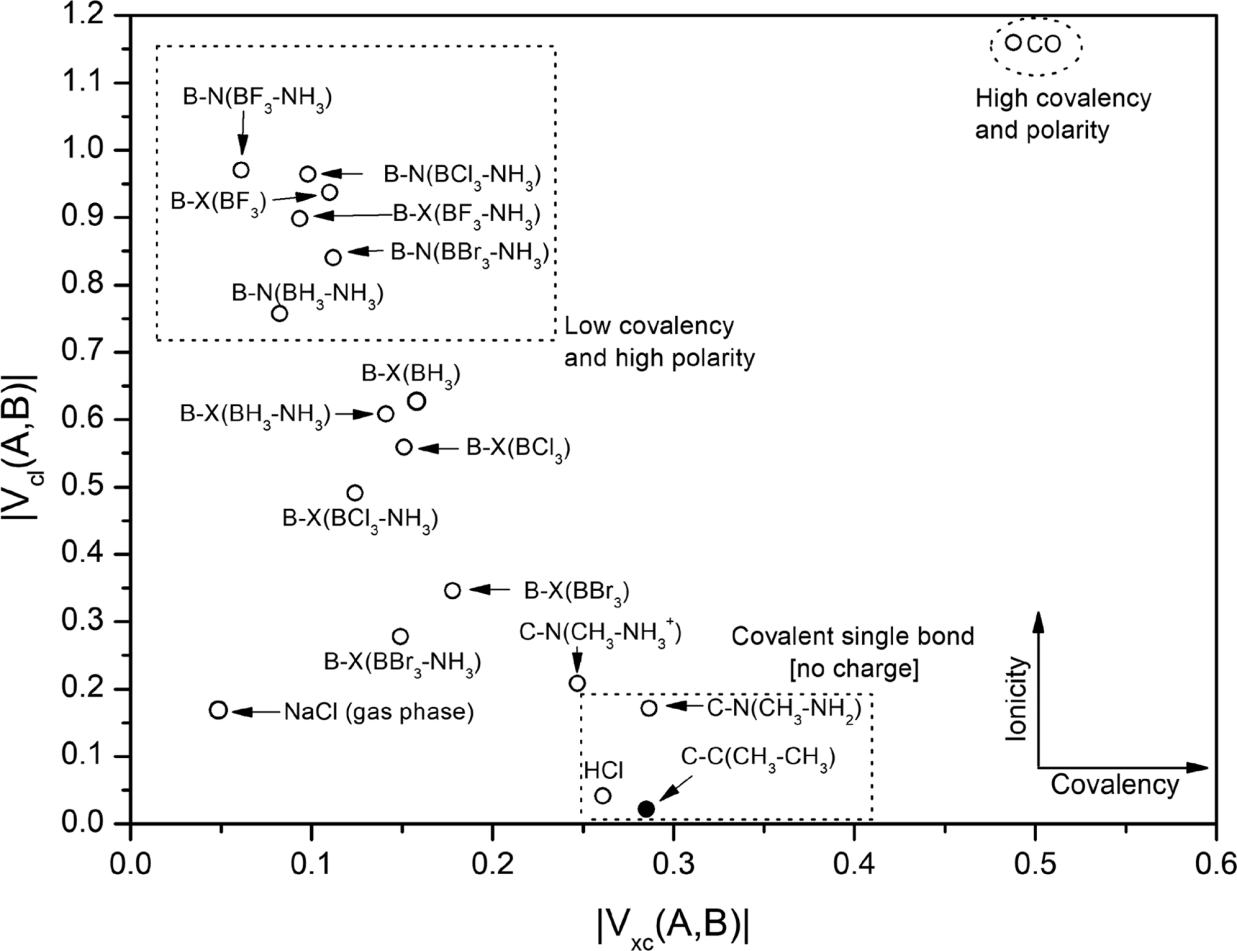
Two-dimensional representation of ionicity [*V*_cl_ (A,B)] against covalency [*V*_xc_ (A,B)] (in Hartree) for B–N and B–X bonds in the free acids and in the complex. NaCl, HCl, and CO were added as reference points. The black dot indicates that *V*_cl_ is positive, namely, the bond is destabilized.

Figure 7 shows a two-dimensional plot of essential ionicity [*V*_cl_ (A,B)] against covalency [*V*_xc_ (A,B)] for B–N and B–X bonds in the free acids and complexes. The ordinate reveals the magnitude of *V*_cl_ (A,B), which is associated with the degree of ionicity. The abscissa displays the magnitude of *V*_xc_ (A,B), which is associated with the degree of covalency. All quantities are displayed in Hartree. Carbon monoxide is an example of a highly covalent molecule that also shows high polarity in its bonds, i.e., ionicity. In contrast, HCl is an example of a single-bond covalent compound where the exchange-correlation contribution is 6 times larger than the classical electrostatic contribution.

The B–F bond is high in ionicity and has only a small contribution from the exchange correlation. This is surprising because the reverse Lewis acidity of boron trihalides has been associated with the π-backbonding effect, which is well accepted by the scientific community and a frequent topic in general undergraduate chemistry courses.

The other B–X bonds also display low covalency and their locations along the *V*_xc_-axis of the 2D plot are close to gas-phase NaCl. This is evidence for the ionic character of the B–X bond according to Gillespie. The covalency/ionicity character of the B–X bond is only slightly affected by the formation of the complex, where the magnitude of *V*_cl_ (A,B) is, on average, reduced by 0.05 au (131 kJ mol^–1^) and the magnitude of *V*_xc_ (A,B) by 0.02 au (53 kJ mol^–1^). As stated before, such changes can be interpreted in terms of the poor overlap of X orbitals with the empty *p* orbital of boron when the acid is rehybridized.

### 4.5 Infrared Analysis and the CCTDP Model

Symmetry arguments show, and quantum calculations confirm, that infrared intensities of out-of-plane bending modes of planar molecules receive no contributions from charge transfer terms. However, upon complex formation, the planar symmetry is broken and the BX_3_ out-of-plane bending mode is modified. Not only is the wavenumber blue- or red-shifted but the intensities change drastically. Earlier work^27^ demonstrates that the enhancement of the hydrogen stretching band that occurs when a hydrogen bond is formed relates to the hydrogen bond energy. Moreover, that work showed that the CCTDP contributions provide useful information on changes in the electronic structure during the formation of the H-bond complex. In the H-bond case, comparisons between monomers and complex intensities were easier since the displacement vector of the hydrogen-bonded H atom and the not-hydrogen-bonded H were similar in magnitude. Now, we are not only comparing the out-of-plane vibration of BX_3_ and the more complicated bending vibration in the BX_3_–NH_3_ complex but also comparing vibrations of different molecules. Note that displacement vectors play an important role in determining the infrared intensities.

Table 3 shows the frequencies (in cm^–1^) and intensities (in km mol^–1^) of the B–X out-of-plane bending for the BX_3_ monomers and the corresponding vibration in the BX_3_–NH_3_ complexes. The C–H out-of-plane bending values are also included for CH_3_CH_3_, CH_3_NH_2_, and [CH_3_NH_3_]^+^. The CCTDP parameters, which are obtained from the square of eq 11, are also reproduced in Table 3. The most electron-deficient molecule (BH_3_) displays the greatest values of C^2^ and DP^2^, which is explained by the mechanical weight (depending on atomic mass) of hydrogens: smaller atoms will show greater displacements, thereby enhancing their contributions.

**Table 3:** Infrared Intensities [km mol^–1^], Frequencies [cm^–1^], and CCTDP Contributions [km mol^–1^] for the Out-of-Plane Bending

molecule	frequency [cm^–1^]	intensity [km mol^–1^]	*C*^2^	*CT*^2^	*DP*^2^	2*CCT*	2*CDP*	2*CTDP*	% *CT*^2 ^a
BH_3_	1174.72	89.53	1701.60	0.00	1010.46	0.00	–2622.52	0.00	0.0
BF_3_	691.33	100.38	680.28	0.00	258.02	0.00	–837.93	0.00	0.0
BCl_3_	459.67	4.94	452.68	0.00	361.78	0.00	–809.37	0.00	0.0
BBr_3_	394.54	0.36	306.26	0.00	285.67	0.00	–591.56	0.00	0.0
BH_3_NH_3_	1209.49	151.64	1590.06	0.73	713.52	–67.90	–2130.22	45.48	0.0
BF_3_NH_3_	686.18	103.18	219.71	0.06	24.46	7.98	–146.39	–2.66	0.0
BCl_3_NH_3_	481.04	14.48	46.91	0.01	9.71	0.94	–42.70	–0.44	0.0
BBr_3_NH_3_	376.47	17.34	80.50	0.43	17.25	–11.69	–74.50	5.40	0.4
CH_3_CH_3_	1416.55	1.12	0.08	188.17	224.11	6.15	–6.72	–410.60	45.6
CH_3_NH_2_	1466.51	2.17	0.12	16.37	13.22	1.67	–2.17	–27.00	55.1
[CH_3_NH_3_]^+^	1481.56	1.51	52.92	6.64	12.1	–37.48	–50.57	17.89	9.3

a %*CT*^2^ = *CT*^2^/(*C*^2^ + *CT*^2^ + *DP*^2^).

Although the *CT* contributions are equal to zero for BX_3_ monomers and are very small for the BX_3_–NH_3_ complexes, charge transfer effects provide important contributions to the intensities of CH_3_CH_3_, CH_3_NH_2_, and [CH_3_NH_3_]^+^. For the methyl substituent group, *CT*^2^ contributes 9–55% of the sum of CCTDP quadratic terms (*C*^2^ + *CT*^2^ + *DP*^2^), while these contributions reach only 0.4% for BX_3_–NH_3_ complexes. In the CCTDP model, charge transfer corresponds to the electrons’ ability to flow from one atom to another when atoms are infinitesimally displaced from the equilibrium position. The electronic density in a covalent bond is easier to deform when the atoms move because it is concentrated between atoms. In ionic bonds, the electronic density is concentrated at the atoms so small displacements will result in a lower charge derivative. The systems that show greater contributions from charge transfer are the ones that present the greater degrees of covalency, as shown in Figure 7. To investigate this further, we have to look at the atomic displacements within the normal coordinate.

**Figure 8:**
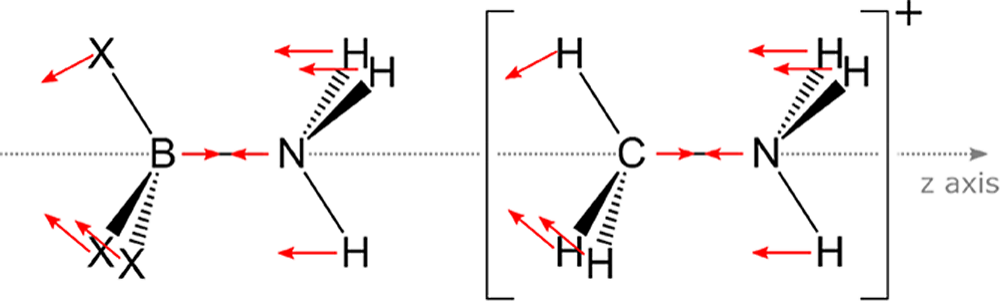
Atomic movements within the out-of-plane bending mode for BX_3_NH_3_ complexes (left) and [CH_3_NH_3_]^+^ (right). Similar movements are found for CH_3_CH_3_ and CH_3_NH_2_. Note the alignment with respect to the *z*-axis. Red arrows indicate the direction of the displacement vectors (magnitude not to scale).

Figure 8 shows the atomic displacement vectors in red, out of scale. Note that for the BX_3_–NH_3_ complex the movement of the atoms mimics the complex formation path, where the X atoms are displaced out of the BX_3_ plane (rehybridization) as the base is approaching. This means that the changes in the electronic density that occur during the vibrational movement are similar to those that occur in the reaction path. A scheme showing the atomic displacements of [CH_3_NH_3_]^+^ is also included to exemplify the similar normal modes of CH_3_CH_3_ and CH_3_NH_2._

When the boron (or carbon) is displaced in the *z* direction, it moves closer (or further from) to the nitrogen atom. The charge transfer that occurs for this movement is measured by the  term of the charge transfer atomic polar tensor (Table 4). For the BX_3_ monomers, these terms are zero. For BX_3_NH_3_ complexes, the magnitude of the *p_zz_* term is the smallest of the three principal diagonal terms (*xx*, *yy*, and *zz*) presenting evidence of the small degree of covalency between B and N, which impedes electron exchange between nuclei. The carbon counterparts exhibit a higher covalency degree between C and N (or C–C). Hence, charge transfers between these nuclei are favored leading to higher values (in magnitude) for the *p_zz_* term relative to the *p_xx_* and *p*_*yy*_ derivatives.

**Table 4:** Charge Transfer Atomic Polar Tensors [e amu^–1/2^] for B or C Atoms Calculated at the MP2/aug-cc-pVTZ Level^a^

BH_3_	–0.98	0.00	0.00	BH_3_NH_3_	–0.96	0.00	0.00	CH_3_CH_3_	–0.44	0.00	0.00
	0.00	–0.98	0.00		0.00	–0.96	0.00		0.00	–0.44	0.00
	0.00	0.00	**0.00**		0.03	–0.01	–**0.49**		0.00	0.00	**0.34**
BF_3_	–0.10	0.00	0.00	BF_3_NH_3_	–0.30	0.00	0.00	[CH_3_NH_3_]^+^	–0.41	0.00	0.00
	0.00	–0.10	0.00		0.00	–0.30	–0.01		0.00	–0.41	0.00
	0.00	0.00	**0.00**		0.00	–0.01	–**0.04**		0.01	0.00	–**1.03**
BCl_3_	–0.61	0.00	0.00	BCl_3_NH_3_	–0.72	0.00	0.00	CH_3_NH_2_	–0.41	0.00	0.00
	–0.01	–0.62	0.00		0.00	–0.72	0.00		0.00	–0.29	0.00
	0.00	0.00	**0.00**		–0.01	0.01	–**0.36**		0.00	0.11	–**1.07**
BBr_3_	–1.40	0.00	0.00	BBr_3_NH_3_	–1.36	0.00	0.00				
	0.00	–1.40	0.00		0.00	–1.36	0.00				
	0.00	0.00	**0.00**		0.00	0.00	–**0.51**				

a Bold values correspond to  terms.

## 5 Conclusions

A detailed IQA study was carried out on the formation of BX_3_–NH_3_ complexes, where X = H, F, Cl, or Br. The complexation reactions were divided into two simpler processes: (i) the acid is allowed to deform (i.e., rehybridize), adopting its pyramidal sp^3^ geometry of the complex and (ii) the acid and base approach each other to form the Lewis acid–base adduct. The IQA analysis on the acid rehybridization reveals that steric effects and classical electrostatics are acting against the adoption of the sp^3^ geometry. The relative energy gradient (REG) analysis over the adduct formation highlighted the main energy components that drive the complexation process, allowing us to understand the energetics behind the formation of the B–N bond. Finally, in the vibrational analysis of the out-of-plane B–X bending, the infrared intensities were decomposed into its atomic charge and dipole derivatives elucidating the electronic density changes with the formation of the adduct.

Although the π-backbonding effect is well accepted in undergraduate-level textbooks, our IQA analysis does not support this explanation. Instead, a REG analysis shows that electrostatic energy terms, not exchange-correlation, explain the energy profile of the chemical path that leads to complex formation. Because π-backbonding and hyperconjugation effects contribute to the exchange-correlation potential energy,^17^ they cannot be supported by REG.

In fact, according to the topological analysis, the B–X bond for the trihalides has a low degree of covalency and its ionicity increases according to the halide’s electronegativity. BF_3_ is the most ionic of the compounds and, therefore, a partial double bond caused by the p(π) overlap is unlikely. The stabilization of the acid–base complex results from a balance between the attractive and repulsive electrostatic energy. An increase in electronegativity of X will increase its charge and thus enhance the magnitudes of both attractive and repulsive energies, resulting in the observed acidity order. The high acidity of BH_3_ relative to that of the boron trihalides does not rely on the equilibrium of repulsive/attractive forces but on its lower rehybridization energy for adopting the pyramidal geometry in the complex. For the trihalides, differences in the rehybridization energies are small.

The IR-CCTDP analysis, performed over the BX_3_ Lewis acids and their adducts, shows that normal modes of vibration are useful to understand electronic structure changes when molecules react. In fact, normal coordinates are obtained following the same procedure to determine reaction coordinates, that is, by finding the eigenvalues of the Hessian matrix. The normal coordinate of the B–X out-of-plane bending defines both the rehybridization process and the base approaching the acid. As a consequence of the systems’ orientation, the *p_zz_* term of the atomic polar tensor is zero for BX_3_ monomers but when the complexes are formed, this term increases with the degree of covalency of the B–N bond.

Since complex formation is dominated by electrostatics, the B–N bond also exhibits a high degree of ionicity. This is confirmed not only by the topological analysis but also by infrared analysis, where the contribution of the charge transfer (%*CT*^2^) between B and N is lower when compared with covalent analogues. The ionic nature of trihalides and their complexes, accompanied by the equilibrium between attractive and repulsive electrostatics, is sufficient to explain the acidity trend in these compounds without an ad hoc explanation.

## Supporting Information Available

Geometrical parameters and QTAIM atomic charges for BX_3_ monomers and BX_3_–NH_3_ complexes (Table S1); and REG analysis results for the reaction path (Table S2) (PDF)
