# Supplementary material for: Electrostatics Explains the Reverse Lewis Acidity of BH3 and Boron Trihalides: Infrared Intensities and a Relative Energy Gradient (REG) Analysis of IQA Energies
Source: J Phys Chem A. 2021 Sep 22;125(39):8615–25. doi: 10.1021/acs.jpca.1c05766 (PMC8503881; doi:10.1021/acs.jpca.1c05766)
Supplement: Supplementary file 1 — jp1c05766_si_001.pdf [file jp1c05766_si_001.pdf]

Supporting Information

Electrostatics explains the reverse Lewis Acidity of BH_3 and Boron Trihalides: Infrared Intensities and a Relative Energy Gradient (REG) Analysis of IQA Energies

Leonardo J. Duarte[a], Wagner E. Richter[b], Roy E. Bruns[a] and Paul L.A. Popelier[c*]

[a]Chemistry Institute, University of Campinas, Campinas, SP, 13083-861, Brazil.

[b]Department of Chemical Engineering, Federal University of Technology - Paraná, Ponta Grossa, PR, 84017-220, Brazil.

[c]Manchester Institute of Biotechnology (MIB), 131 Princess Street, Manchester M1 7DN, Great Britain and Department of Chemistry, University of Manchester, Oxford Road, Manchester M13 9PL, Great Britain

Table S1. Geometrical parameters and QTAIM atomic charges for BX_3 monomers and BX_3-NH_3 complexes. Equilibrium charges (q°) and bond distances (r) are given in units of *e* and Å, respectively.

	q° (B)	q° (X)	q° (N)	r (B-X)	r (B-N)
			**Monomer**		
**BH_3**	2.03	-0.68	--	1.18	--
**BF_3**	2.53	-0.84	--	1.32	--
**BCl_3**	2.15	-0.72	--	1.74	--
**BBr_3**	1.81	-0.60	--	1.89	--
			**Complex**		
**BH_3**	1.98	-0.68	-1.21	1.20	1.65
**BF_3**	2.51	-0.87	-1.24	1.37	1.67
**BCl_3**	2.15	-0.74	-1.30	1.82	1.61
**BBr_3**	1.85	-0.63	-1.32	1.98	1.60

Table S2. REG analysis results for the reaction path. The control coordinate is the B-N distance. Segment 1 corresponds to the shortening of the B-N bond beyond the equilibrium point, while Segment 2 corresponds to the formation of B-N bond. The atom X corresponds to H, F, Cl and Br.

Acid	Segment 1			Segment 2		
	**IQA Term**	**REG**	**R^2**	**IQA Term**	**REG**	**R^2**
BH_3	$E_{intra}(N)$	1.31	0.90	$V_{cl}(B,N)$	9.36	0.95
	$V_{cl}(B,X)$	1.07	0.96	$V_{cl}(X,H)$	6.98	0.93
	$V_{cl}(X,H)$	0.34	1.00	$V_{cl}(N,H)$	3.08	0.99
	$E_{intra}(B)$	0.27	0.88	$V_{xc}(X,N)$	1.67	1.00
	$V_{cl}(B,H)$	0.22	0.44	$V_{xc}(B,N)$	1.61	0.96
	$V_{xc}(X,N)$	−0.20	0.86	$V_{xc}(N,H)$	−1.02	1.00
	$E_{intra}(X)$	−0.27	0.92	$E_{intra}(H)$	−1.10	0.99
	$V_{xc}(B,N)$	−0.31	0.92	$E_{intra}(N)$	−2.58	0.92
	$V_{cl}(X,X)$	−0.33	0.94	$V_{cl}(X,N)$	−6.97	0.91
	$V_{cl}(B,N)$	−1.12	0.67	$V_{cl}(B,H)$	−7.93	0.96
BF_3	$E_{intra}(N)$	1.48	0.90	$V_{cl}(B,N)$	16.28	0.98
	$V_{cl}(B,X)$	0.96	0.91	$V_{cl}(X,H)$	12.24	0.98
	$V_{cl}(B,H)$	0.88	0.82	$V_{cl}(N,H)$	5.30	0.96
	$V_{cl}(X,N)$	0.48	0.73	$V_{xc}(X,N)$	2.73	0.99
	$E_{intra}(B)$	0.17	0.89	$E_{intra}(B)$	2.39	0.85
	$E_{intra}(X)$	−0.23	0.83	$E_{intra}(H)$	−1.80	0.98
	$V_{cl}(X,X)$	−0.24	0.89	$V_{cl}(B,X)$	−2.13	0.44
	$V_{xc}(B,N)$	−0.25	0.92	$E_{intra}(N)$	−3.73	0.86
	$V_{cl}(N,H)$	−0.83	0.89	$V_{cl}(X,N)$	−11.89	0.96
	$V_{cl}(B,N)$	−1.49	0.71	$V_{cl}(B,H)$	−14.17	0.99
BCl_3	$E_{intra}(N)$	1.49	0.91	$V_{cl}(B,N)$	15.96	0.95
	$V_{cl}(B,X)$	0.86	0.97	$V_{cl}(X,H)$	9.03	0.88
	$V_{cl}(B,H)$	0.67	0.81	$V_{cl}(N,H)$	7.66	0.99
	$E_{intra}(B)$	0.31	0.83	$V_{xc}(B,N)$	2.78	0.99
	$V_{cl}(X,N)$	0.18	0.51	$V_{xc}(X,N)$	2.62	0.94
	$V_{cl}(X,X)$	−0.21	0.94	$E_{intra}(H)$	−2.45	0.99
	$E_{intra}(X)$	−0.23	0.89	$V_{cl}(B,X)$	−5.72	0.95
	$V_{xc}(B,N)$	−0.25	0.94	$E_{intra}(N)$	−5.82	0.95
	$V_{cl}(N,H))$	−0.75	0.88	$V_{cl}(X,N)$	−8.76	0.88
	$V_{cl}(B,N)$	−1.41	0.75	$V_{cl}(B,H)$	−12.31	0.95
BBr_3	$E_{intra}(N)$	1.57	0.92	$V_{cl}(B,N)$	14.87	0.94
	$V_{cl}(B,H)$	0.73	0.84	$V_{cl}(N,H)$	8.50	0.99
	$E_{intra}(B)$	0.53	0.88	$V_{cl}(X,H)$	7.18	0.81
	$V_{cl}(B,X)$	0.41	0.98	$V_{xc}(B,N)$	3.44	0.97
	$V_{xc}(B,X)$	0.21	0.86	$E_{intra}(X)$	3.04	0.98
	$E_{intra}(X)$	−0.12	0.86	$E_{intra}(H)$	−2.68	0.96
	$V_{xc}(X,N)$	−0.16	0.86	$E_{intra}(N)$	−6.62	0.96
	$V_{xc}(B,N)$	−0.27	0.94	$V_{cl}(X,N)$	−6.69	0.81
	$V_{cl}(N,H)$	−0.77	0.89	$V_{cl}(B,X)$	−6.93	0.94
	$V_{cl}(B,N)$	−1.50	0.77	$V_{cl}(B,H)$	−10.87	0.92
